# Integrated Microbiome and Metabolomics Analysis Reveals Altered Aggressive Behaviors in Broiler Chickens Showing Different Tonic Immobility

**DOI:** 10.3390/ani15040601

**Published:** 2025-02-19

**Authors:** Jiang Gao, Xiaoxian Cheng, Xuanfu Wu, Cunzhi Zou, Bin He, Wenqiang Ma

**Affiliations:** 1 Key Laboratory of Animal Physiology and Biochemistry, Ministry of Agriculture and Rural Affairs, College of Veterinary Medicine, Nanjing Agricultural University, Nanjing 210095, China; 2022007052@stu.njau.edu.cn (J.G.); cv15550809576@163.com (X.C.); 2021807150@stu.njau.edu.cn (X.W.); 18011590230@163.com (C.Z.); heb@njau.edu.cn (B.H.); 2 MOE Joint International Research Laboratory of Animal Health & Food Safety, Nanjing Agricultural University, Nanjing 210095, China

**Keywords:** tonic immobility, broiler chickens, aggressive behavior, microbiome, metabolomics

## Abstract

Tonic immobility (TI) serves as an indicator of innate stress response recovery in poultry. Broilers with different TI phenotypes exhibit varying levels of aggressive behavior, which can significantly impact their welfare. However, the influences of TI phenotypes on broiler aggression remain largely unexplored. In this study, broiler chickens were stratified into two distinct phenotypic groups based on the TI duration: short TI (STI) and long TI (LTI). The impacts of TI phenotypes on broiler aggression were investigated by analyzing cecal intestinal morphology, cecal bacteria, plasma metabolites, and corticosterone levels. Compared to LTI broilers, STI broilers showed significantly reduced plasma corticosterone (CORT) levels (*p* < 0.05) and a decreased frequency of aggressive behaviors, including dominant and subdominant types (*p* < 0.01). Histological analysis revealed that STI broilers have an increased duodenal villus height and villus-height-to-crypt-depth ratio (*p* < 0.01), a decreased jejunal crypt depth with an increased villus-height-to-crypt-depth ratio (*p* < 0.01), and a reduced ileal crypt depth and villus height (*p* < 0.01) compared to LTI broilers. 16S rDNA sequencing and Linear discriminant analysis effect size (LefSe) identified differential cecal bacterial abundance, notably in the genus *cc115* belonging to Firmicutes. Specific microbiota in LTI broilers exhibited significant positive correlations with aggressive behavior and plasma corticosterone, while those in STI broilers showed significant negative correlations. Untargeted plasma metabolomics revealed 21 downregulated and 17 upregulated metabolites between TI phenotypes. Correlation analysis showed that the genus *cc115* and 10 plasma metabolites were positively correlated with aggressive behavior, whereas 8 metabolites were negatively correlated. LTI broilers have higher plasma corticosterone content and more intense aggressive behavior than STI broilers. The distinct behavioral and physiological profiles observed in broilers with different TI phenotypes are strongly correlated with their specific gut microbiota and differential plasma metabolite profiles. The identified gut microbial signatures serve as key biomarkers for regulating aggressive behavior in broilers, while the differential plasma metabolites represent potential early indicators for detecting stress and behavioral issues in poultry farming.

## Simple Summary

This study explored the relationship between tonic immobility (TI) phenotypes and aggressive behavior in broiler chickens. We integrated microbiome and metabolomics analyses to reveal that broilers with short TI (STI) exhibit lower plasma corticosterone levels and reduced aggressive behaviors compared to those with long TI (LTI). The research identified specific gut microbiota and plasma metabolites that correlate with aggressive behavior, highlighting potential biomarkers for improving broiler welfare and productivity. These findings suggest that modulating gut microbiota and monitoring plasma metabolites could be effective strategies to manage stress and aggression in poultry farming.

## 1. Introduction

TI is an evolutionarily conserved instinctual response across animals, characterized by transient akinesis and muscular rigidity following significant environmental stimuli. TI serves as a metric for evaluating fear levels, with prolonged episodes often indicative of heightened fearfulness [1,2]. In broiler chickens, TI is not only an innate fear response but also a critical welfare indicator, reflecting the animal’s ability to cope with stressors. Broilers can be classified into LTI and STI phenotypes based on their latency to resume mobility after induction. These phenotypes are associated with distinct behavioral and physiological characteristics, where STI chickens show superior growth performance, stress adaptability, and welfare outcomes, while LTI chickens are more susceptible to chronic stress, reduced growth, poor feed efficiency, and heightened disease risk [3,4].

Aggressive behavior is another significant factor influencing the welfare and productivity of broiler chickens. Various environmental and physiological factors, including stocking density, lighting schedules, and stress exposure, can drive aggression in broilers [5]. Among these, CORT, the primary glucocorticoid in birds, plays a pivotal role in stress responses and has been closely linked to TI. LTI broilers typically exhibit elevated plasma CORT levels compared to STI broilers [6,7,8]. In addition, elevated CORT levels have been directly associated with increased aggression, potentially through alterations in neurotransmitter systems and stress-responsive pathways [9].

Beyond CORT, recent studies highlight the critical role of the gut microbiome and plasma metabolites in regulating behavior and stress responses in broilers. The gut microbiome, a key component of gastrointestinal health, has emerged as a central regulator of host behavior through the gut–brain axis. Distinct TI phenotypes have been correlated with unique gut microbial compositions, indicating that microbiota may influence fear responses and stress coping mechanisms [10]. Transplantation of cecal microbiota has been shown to reduce aggression by altering the gut microbial composition and influencing the central serotonergic and catecholaminergic systems in recipient chickens [11,12]. Furthermore, microbial metabolites and plasma metabolites have been implicated in behavioral modulation through various pathways, including neurotransmitter regulation, hypothalamic–pituitary–adrenal (HPA) axis activation, and synaptic plasticity adjustments [13,14]. For instance, alterations in hypothalamic serotonin (5-HT) levels have been linked to plasma tryptophan levels; tryptophan is a precursor for serotonin synthesis, and its levels in plasma are inversely correlated with aggressive behavior, suggesting a potential role of serotonergic and dopaminergic systems in modulating TI and aggressive behaviors [15,16].

Despite these findings, the relationship between gut microbiota, plasma metabolites, and aggressive behavior in broilers with different TI phenotypes remains poorly understood. This study aimed to integrate microbiome and metabolomics analyses to investigate the effects of cecal microbiota and plasma metabolites on aggressive behavior in broilers exhibiting distinct TI phenotypes. By elucidating the complex interactions between gut microbes, metabolites, and behavior, the findings of this study are expected to provide novel strategies for improving animal welfare and productivity in broiler farming.

## 2. Materials and Methods

### 2.1. Animals and Experimental Design

Six hundred healthy, one-day-old Partridge shank broiler chickens (fast-growing commercial strain of broiler chickens in China with a rapid growth rate, efficient feed conversion, and consistent performance) were housed in cages measuring 90 cm × 60 cm × 40 cm (length × width × height) within a temperature-controlled animal room at Nanjing Agricultural University, with unrestricted access to food and water, and subjected to a daily light cycle of 16 h of light and 8 h of darkness, all chickens sharing a room with a temperature of 25 ± 2 °C and 60–65% relative humidity. The initial stocking density was established at 50 broilers per cage until day 7, subsequently adjusted to 20 broilers per cage from days 8 to 21, and finally reduced to 10 broilers per cage from day 22 through day 37. To define TI phenotypes, we developed two separate models: short tonic immobility (STI) and long tonic immobility (LTI). To segregate the broilers into these two groups, TI induction trials were carried out at day 18 and day 26, respectively. Subsequent data collection included body weights at 28 and 35 days of age. Individual identification was facilitated via leg bands, with each experimental group comprising 50 chickens. The assessment of TI in the poultry subjects was carried out following the protocol delineated by Jones R.B. [17]. The chickens were relocated to an isolated, tranquil space with subdued illumination. The researchers positioned each bird supine on a level surface, exerting slight pressure on the thoracoabdominal region, while concurrently stabilizing the head gently. This approach typically induced a state of total muscular rigidity within seconds, initiating the timing sequence. The operator then disengaged and receded to an appropriate distance, taking care to limit motion and sound to avert any undue disturbance or visual interaction with the avian subject.

A successful induction of TI was noted if the broiler remained in the supine position for a minimum of 10 s, with the duration being noted until the bird autonomously righted itself. In instances where the broiler remained in TI for over 3 min, the duration was capped and recorded as 180 s. Conversely, if the broiler self-righted within 10 s, the induction was recorded as unsuccessful, warranting a repeat of the procedure. If induction failed three times consecutively, the TI duration was recorded as zero seconds. Each tester had been specially trained to unify the operation and judgment standards. The experiment recorded broilers exhibiting the shortest and longest durations of TI. Broilers were divided into two groups: 50 with the shortest TI duration formed the STI group, and 50 with the longest duration formed the LTI group, for a total of 100 broilers, a total of 10 cages, and 5 cages in each group. Chickens with an intermediate TI duration were excluded from the study.

### 2.2. Aggression Experiment

Aggressive behavioral assays were carried out at 35–36 days of age within a designated area isolated from the broiler housing facility. The assay was conducted in an enclosure mirroring the specifications of the broilers’ growth habitat. For the assay, first, we conducted a pre-experiment and found that there was no significant difference in the impact of capture time on the TI duration; second, when we conducted the TI test, a total of six trained professionals conducted the test at the same time to ensure that the test was completed within 3 h. Each tested broiler was randomly selected from each repetition of each group, and the broilers were randomly sampled from each cohort and were individually marked with non-toxic colored dyes for subsequent identification. Pairs of broilers, each sourced from the same cohort but unfamiliar to each other and housed separately, were introduced concurrently into the test enclosure. The interactions were documented over a 60 min interval using video surveillance equipment. Following each session, a new pair was introduced for observation. Throughout the video recording phase, researchers remained at least 1 m distant from the test apparatus. Video capture was facilitated by a quartet of cameras, each positioned atop the enclosures and interfaced with computer systems, enabling comprehensive recording for later analytical review. This behavioral study was conducted over a 2-day span with designated testing intervals from 8:00 to 11:00 and 14:00 to 17:00. Subsequent to the video documentation, three experimenters undertook a comprehensive review and analysis of the recorded material. They collated data on the frequency of agonistic behaviors exhibited by each group of broilers during the 60 min observation period. The characterization of agonistic behavior was based on the methodology ascribed by Kitaysky [6] in Table S1.

### 2.3. Sample Collection

At 37 days of age, 2 chickens were randomly selected from each replicate in each group, with a total of 10 chickens in each group, the broilers were slaughtered by cervical dislocation after blood samples were obtained, a total of 20 chickens were sacrificed. Blood samples from the external jugular vein were immediately transferred into heparin sodium tubes and centrifuged at 3500 rpm and 4 °C for 10 min to separate the plasma. Intestinal tissue samples were preserved in 10% phosphate-buffered formalin for H&E staining examination. Frozen plasma and cecal content samples were stored at −80 °C, and tissue samples were stored at 4 °C for subsequent analysis.

### 2.4. High-Throughput Sequencing of Cecal Microbiota

For preservation of broiler cecal samples for genomic analysis, 18 cecal content specimens from broilers (nine replicates per group) were rapidly cryopreserved in liquid nitrogen and subsequently stored at −80 °C pending DNA extraction. Bacterial 16S rDNA gene hypervariable regions (V3–V4) were amplified using genomic DNA as a template and a primer pair (forward primer ACTCCTACGGGAGGCAGCA, reverse primer GGACTACHVGGGTWTCTAAT) in a polymerase chain reaction. Real-time PCR was conducted on the cDNA product using Applied Biosystems procedures. The selected specimens were sequenced using the Illumina MiSeq PE300 platform (San Diego, CA, USA) by Panomix Biomedical Technology Company (Suzhou, China).

### 2.5. Plasma Biochemical Parameter Analysis

Plasma concentrations of Aspartate aminotransferase AST (H002), Alanine aminotransferase ALT (H001), Total protein TP (H102), Albumin ALB (H103T), Total bile acid TBA (H101T), Glucose GLU (H108), Calcium Ca (H303), Cholesterol CHOL (H202), Triglyceride TG (H201), High density lipoprotein cholesterol HDL-C (H203T), Low density lipoprotein LDL-C (H207), Lactate dehydrogenase LDH (H008), and Non-esterified fatty acids NEFA (D444) were measured using a Beckman Coulter AU2700 automatic biochemical analyzer (Beckman, California, USA) with commercial kits from MALLBIO (Nanjing, China). Plasma CORT concentrations were determined using commercial ELISA kits (E-EL-0160c) from Elabscience (Wuhan, China) following the manufacturer’s instructions.

### 2.6. Metabolomic Profiling of Plasma

Metabolomics analysis of plasma samples was conducted by Suzhou Panomix Biomedical Technology Company. The samples were thawed at 4 °C and vortexed for 1 min to ensure thorough mixing. An exact portion of the sample was transferred into a 2 mL centrifuge tube. Subsequently, 400 µL of methanol (pre-cooled to −20 °C) was added, and the mixture was vortexed again for 1 min. The sample underwent centrifugation at 12,000 rpm for 10 min at 4 °C. After centrifugation, the supernatant was carefully transferred to a fresh 2 mL centrifuge tube, concentrated, and dried. The dried sample was reconstituted with 150 µL of a 4 ppm 2-chloro-L-phenylalanine solution in 80% methanol and stored at 4 °C. The supernatant was filtered using a 0.22 μm membrane and placed into a sample vial for LC-MS analysis. A Vanquish UHPLC system (Thermo Fisher Scientific, MA, USA) with an ACQUITY UPLC^®^ HSS T3 column (150 mm × 2.1 mm, 1.8 µm; Waters, Milford, MA, USA) was used for liquid chromatography analysis. An Orbitrap Exploris 120 system (Thermo Fisher Scientific, MA, USA) equipped with an electrospray ionization (ESI) source was used for mass spectrometric analysis. Both MS1 and MS/MS data were acquired simultaneously using full MS-ddMS2 mode (data-dependent MS/MS). The MS conditions included a sheath gas flow rate of 30 arb, an auxiliary gas flow rate of 10 arb, spray voltages of 3.50 kV for ESI (+) and −2.50 kV for ESI (−), and a capillary temperature of 325 °C. The MS1 scan range was *m*/*z* 100–1000 with a resolving power of 60,000 FWHM. A maximum of four data-dependent scans per cycle were conducted, with the MS/MS resolving power configured at 15,000 FWHM. The normalized collision energy was 30%, and the dynamic exclusion time was automatically adjusted.

### 2.7. Statistical Analysis

Data were presented as the mean ± standard error of the mean (SEM). All statistical analyses were performed using IBM SPSS Statistics 20.0. Between-group comparisons were conducted using an independent two-tailed Student’s *t*-test after verification of normality (Shapiro–Wilk test, *p* > 0.05) and homogeneity of variance (Levene’s test, *p* > 0.05). Statistical significance was defined as *p* < 0.05. Bioinformatic analysis used OmicStudio tools (https://www.omicstudio.cn/tool, accessed on 20 June 2024).

## 3. Results

### 3.1. Effects of Different TI on the Production Performance and Aggressive Behaviors of Broilers

**Figure 1:**
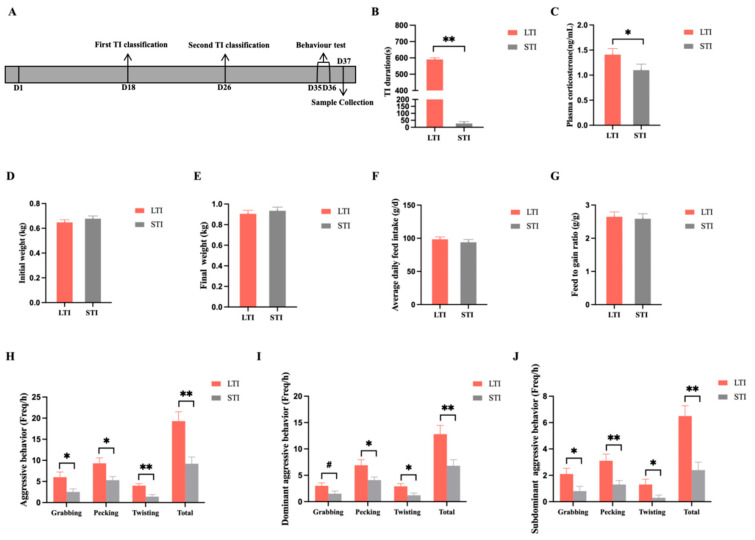
Effects of different TI on the apparent performance of broilers. (**A**) Timeline of the experiment. (**B**) TI duration. (**C**) Plasma corticosterone (mg/mL). (**D**) Initial weight (kg). (**E**) Final live weight (kg). (**F**) Average daily feed intake (g/d). (**G**) Feed-to-gain ratio (g/g). (**H**) Aggressive behavior (Freqs/h). (**I**) Dominant aggressive behavior (Freqs/h). (**J**) Subdominant aggressive behavior (Freqs/h). All data are shown as the mean ± SEM; # *p* < 0.10, * *p* < 0.05, ** *p* < 0.01, n = 10.

The main timeline of the experiment is shown in Figure 1A. STI broilers demonstrated a significant reduction in the TI duration (*p* < 0.05, Figure 1B) and a notable decrease in the plasma corticosterone content (*p* < 0.05, Figure 1C) compared to LTI broilers. Compared to LTI broilers, STI broilers exhibited a lower frequency of aggressive behavior (*p* < 0.01, Figure 1H), including grabbing, pecking, and twisting; this trend was consistent among dominant and subdominant broilers, where STI broilers showed a significantly lower incidence of aggression as well (*p* < 0.01, Figure 1I,J), including grabbing, pecking, and twisting. A downward trend in grabbing was observed in dominant broilers (*p* < 0.10).

### 3.2. Effects of Different TI on Plasma Biochemical Indexes

Significant differences were observed in the plasma biochemical profiles between the STI and LTI broilers, with the STI group exhibiting notably lower concentrations of AST, TP, Ca, and NEFA compared to their LTI counterparts (*p* < 0.05, Table S1).

### 3.3. Effects of Different TI on the Intestinal Morphology of Broilers

**Figure 2:**
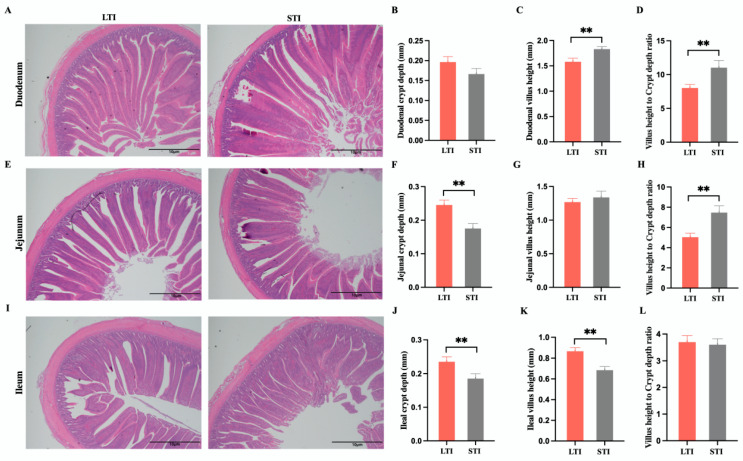
Effects of different TI on the small-intestinal development and morphology of broilers. (**A**) H&E staining of the duodenum (scale bars = 10 μm). (**B**) Duodenal crypt depth (mm). (**C**) Duodenal villus height (mm). (**D**) Duodenal villus-height-to-crypt-depth ratio. (**E**) H&E staining of the jejunum (scale bars = 10 μm). (**F**) Jejunal crypt depth (mm). (**G**) Jejunal villus height (mm). (**H**) Jejunal villus-height-to-crypt-depth ratio. (**I**) H&E staining of the ileum (scale bars = 10 μm). (**J**) Ileal crypt depth (mm), (**K**) Ileal villus height (mm). (**L**) Ileal villus-height-to-crypt-depth ratio. All data are shown as the mean ± SEM; ** *p* < 0.01, n = 3.

A representative photomicrograph of H&E-stained small-intestine sections of broiler chickens is shown in Figure 2. The difference in intestinal morphology has an important impact on the nutrient absorption and metabolite changes of broilers. The duodenum morphology (µm) of chickens is shown in Figure 2A. Compared to LTI broilers, the villus height and villus-height-to-crypt-depth ratio showed a significant increase in STI broilers (*p* < 0.01, Figure 2C,D). The jejunum morphology (µm) of chickens is shown in Figure 2E. A significant decrease in the crypt depth and a significant increase in the villus-height-to-crypt-depth ratio were found in STI broilers compared to LTI broilers (*p* < 0.01, Figure 2F,H). The ileum morphology (µm) of chickens is shown in Figure 2I. The crypt depth and villus height of STI broilers were lower than those of LTI broilers (*p* < 0.01, Figure 2J,K).

### 3.4. Effects of Different TI on the Cecal Microbiota of Broilers

**Figure 3:**
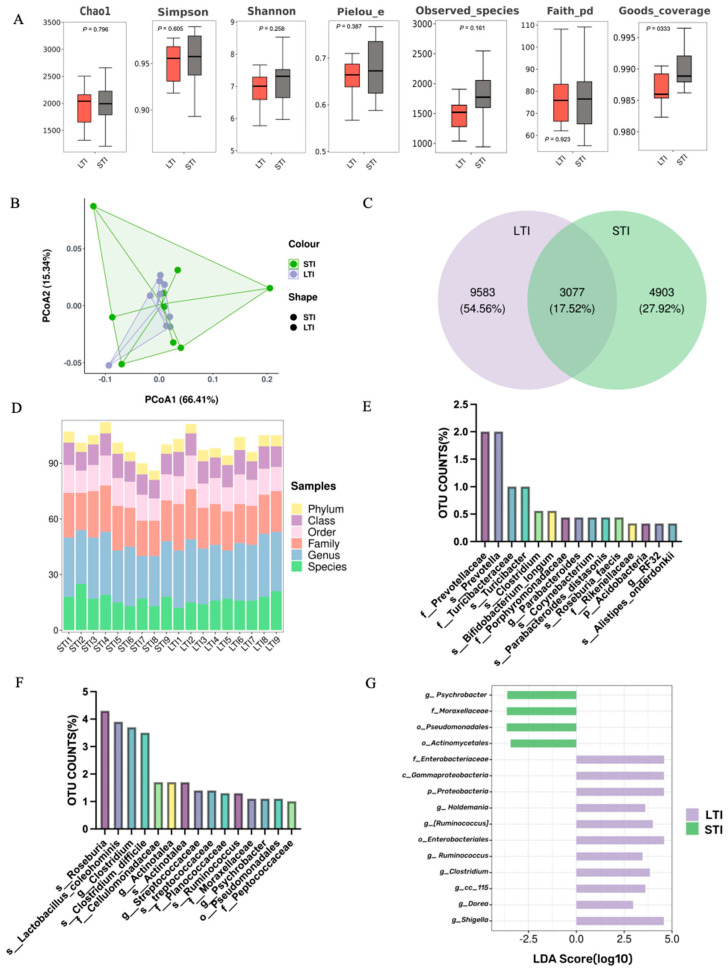
Effects of different TI on the cecal microbiota of broilers. (**A**) Cecal content microbial α-diversity. (**B**) Principal coordinate analysis (PCoA) based on total OTUs. (**C**) Venn diagram based on the total number of OTUs. (**D**) Relative abundance levels of cecal microbiota in different TI phenotypes. (**E**) Specific cecal microbiota of LTI broilers. (**F**) Specific cecal microbiota of STI broilers. (**G**) A histogram of LDA scores for differentially abundant taxa. All data are shown as the mean ± SEM; n = 9.

Figure 3A indicates no statistically significant difference in alpha diversity (*p* > 0.05) and the alpha diversity indexes, including chao1, simpson, shannon, pielou_e, observed_species, faith_pd, and goods coverage between the LTI and STI broilers. Principal coordinates analysis (PCoA) revealed difference degrees based on both unweighted UniFrac and Bray–Curtis dissimilarity metrics (Figure 3B). Figure 3C shows a 17.52% difference in OTUs between the LTI and STI broilers and 54.56% specific OTUs in LTI broilers; however, there were only 27.92% specific OTUs in STI broilers. The proportional representation of different microbial abundance levels within each sample is shown in Figure 3D. The top 15 specific cecal microbiota identified in LTI broilers are shown in Figure 3E, with higher OTU counts of cecal microbiota, including *f_Prevotellaceae*, *s_Prevotella*, *f_Turicibacteraceae*, *s_Turicibacter*, and *s_Clostridium*. Similarly, the top 15 specific cecal microbiota identified in STI broilers are shown in Figure 3F, with higher OTU counts of cecal microbiota, including *s_Roseburia*, *s_Lactobacillus_coleohominis*, *g_Clostridium*, *s_Clostridium_difficile*, and *f_Cellulomonadaceae*. The dominant strains within different TI groups showed significant differences. LEfSe analysis identified the top 15 cecal microbiota in both groups, with high LDA scores, as shown in Figure 3G. In the STI broilers, *g_Psychrobacter*, *f_Moraxellaceae*, *o_Pseudomonadales*, and *o_Actinomycetales* were predominant, while the LTI group was dominated by the remaining 11 microbiota, including *f_Enterobacteriaceae*, *c_Gammaproteobacteria*, *p_Proteobacteria*, *o_Enterobacteriales*, and *g_Shigella*. The dominant strains and the number of shared strains in different groups were significantly different.

### 3.5. Effects of Different TI on the Plasma Metabolites of Broilers

**Figure 4:**
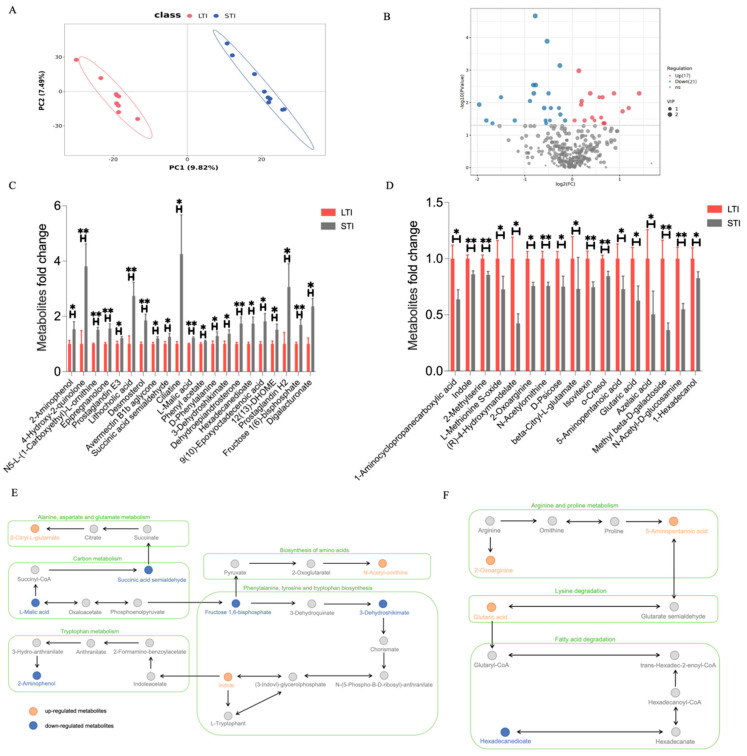
Effects of different TI on the plasma metabolites of broilers. (**A**) Principal component analysis (PCA) results of different TI phenotypes. (**B**) Volcano map showing the differential metabolites between LTI and STI broilers. (**C**) Significant upregulated differential metabolites. (**D**) Significant downregulated differential metabolites. (**E**,**F**) Partial differential metabolite enrichment pathways. All data are shown as the mean ± SEM; * *p* < 0.05, ** *p* < 0.01, n = 9.

Principal component analysis (PCA) was conducted on the samples requiring differential analysis to assess intergroup variability. Figure 4A illustrates the two-dimensional spatial distribution of principal components derived from the metabolic profiling analysis. Principal component analysis (PCA) revealed distinct clustering patterns between the experimental groups, demonstrating significant metabolic divergence. To further refine this discrimination, we used orthogonal partial least squares discriminant analysis (OPLS-DA), which enabled the identification of 38 statistically significant differential metabolites (variable importance in projection (VIP) > 1 and *p* < 0.05). These differentially expressed metabolites were subsequently visualized in a volcano plot (Figure 4B), providing a comprehensive overview of the metabolic shifts between the two groups. Among the 17 upregulated metabolites, key compounds exhibiting significant elevation (fold change > 2) included 4-hydroxy-2-quinolone, lithocholic acid, ciliatine, and prostaglandin H2 (Figure 4C); additionally, 21 metabolites were downregulated, including (R)-4-hydroxymandelate, azelaic acid, and methyl beta-D-galactoside, with a fold change below 0.5 (Figure 4D). A pie chart showed the classification of all 38 differential metabolites, with the largest proportion being of fatty acyls (Figure S1). To elucidate the biological significance of the identified differential metabolites, we performed pathway enrichment analysis using the Kyoto Encyclopedia of Genes and Genomes (KEGG) database. Correlation network diagrams were created using the KEGG pathway database to explore latent relationships among the main enriched pathways. Functional enrichment analysis identified several major metabolic pathways with significant alterations (*p* < 0.05, FDR < 0.1), including alanine, aspartate, and glutamate metabolism (ko00250); carbon metabolism (ko01200); amino acid biosynthesis (ko01230); tryptophan metabolism (ko00380); and phenylalanine, tyrosine, and tryptophan biosynthesis (ko00400) (Figure 4E,F). These findings suggest that metabolic perturbations primarily affect core amino acid metabolic pathways, potentially influencing neurotransmitter synthesis and energy metabolism.

### 3.6. Correlation Analysis Between Aggressive Behavior, Plasma Corticosterone, Same Differential Cecal Microbiota, and Differential Plasma Metabolites

**Figure 5:**
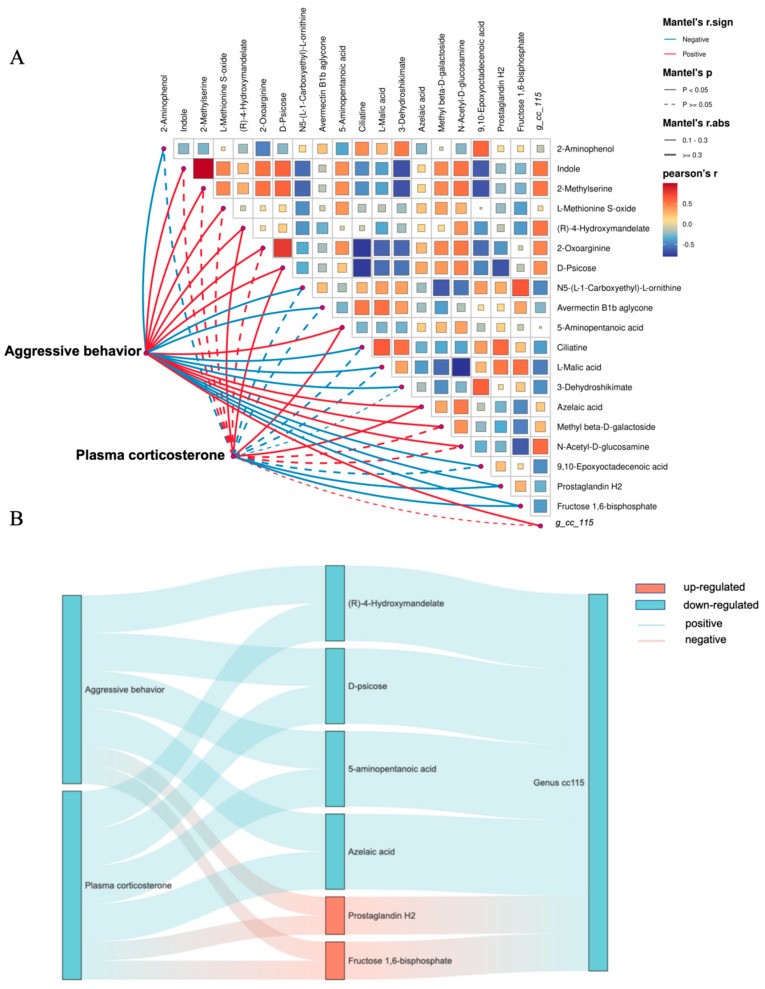
Correlation analysis between aggressive behavior, plasma corticosterone, same differential cecal microbiota, and differential plasma metabolites. (**A**) Correlation network heatmap of aggressive behavior, plasma corticosterone, differential common bacteria, and metabolites. (**B**) Sankey diagram of aggressive behavior, plasma corticosterone, key bacteria, and metabolites.

To explore the intricate relationships between behavioral phenotypes, endocrine markers, and microbial signatures, we constructed a correlation network heatmap through integrated analysis using Mantel tests and Pearson’s correlation coefficients (Figure 5A). The levels of 19 differential metabolites were strongly correlated with aggressive behavior, and genus cc115 was the only one of the differential cecal microbiota correlated with aggressive behavior (Figure S2). This genus-level specificity suggests a potential role of cc115 in mediating behavioral phenotypes through microbial–host interactions, possibly via metabolic or immune modulation. As shown in Figure 5B, aggressive behavior showed a negative correlation with (R)-4-hydroxymandelate, D-psicose, 5-aminopentanoic acid, and azelaic acid, the same relationship as with genus cc115, while aggressive behavior showed a positive correlation with prostaglandin H2 and fructose 1,6-diphosphate, and these metabolites were positive correlated with plasma corticosterone and genus cc115 as well.

### 3.7. Correlation Analysis Between Aggressive Behavior, Plasma Corticosterone, Specific Differential Cecal Microbiota, and Differential Plasma Metabolites

**Figure 6:**
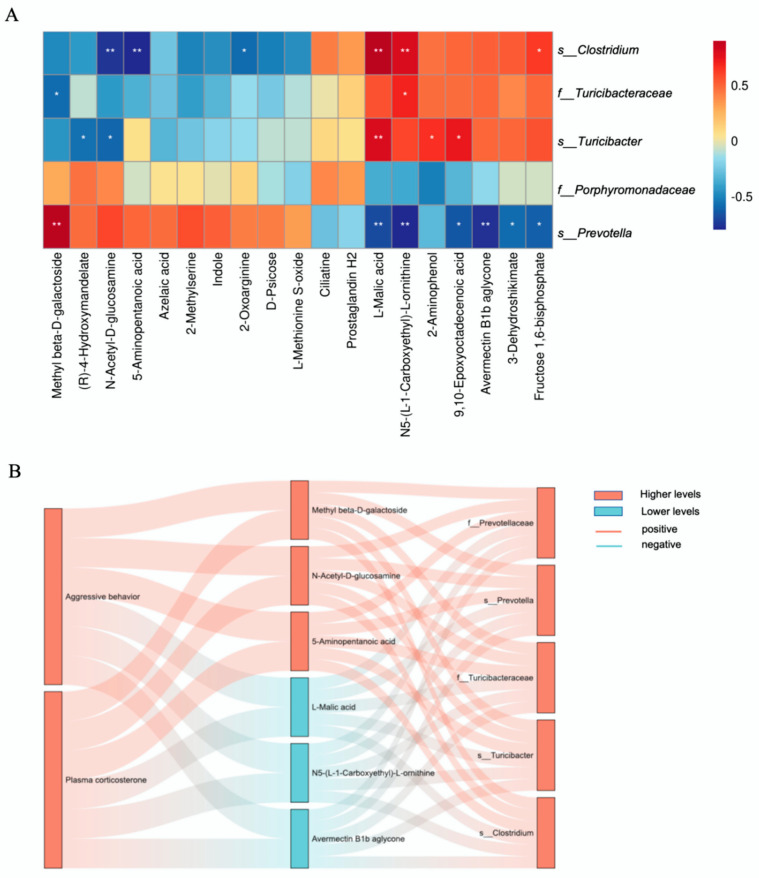
Specific differences correlation analysis in LTI broilers. (**A**) Heatmap of top 5 specific bacteria in LTI broilers and differential metabolites. (**B**) Sankey diagram of aggressive behavior, plasma corticosterone, top 5 bacteria, and differential metabolites in LTI broilers. * *p* < 0.05, ** *p* < 0.01, n = 9.

**Figure 7:**
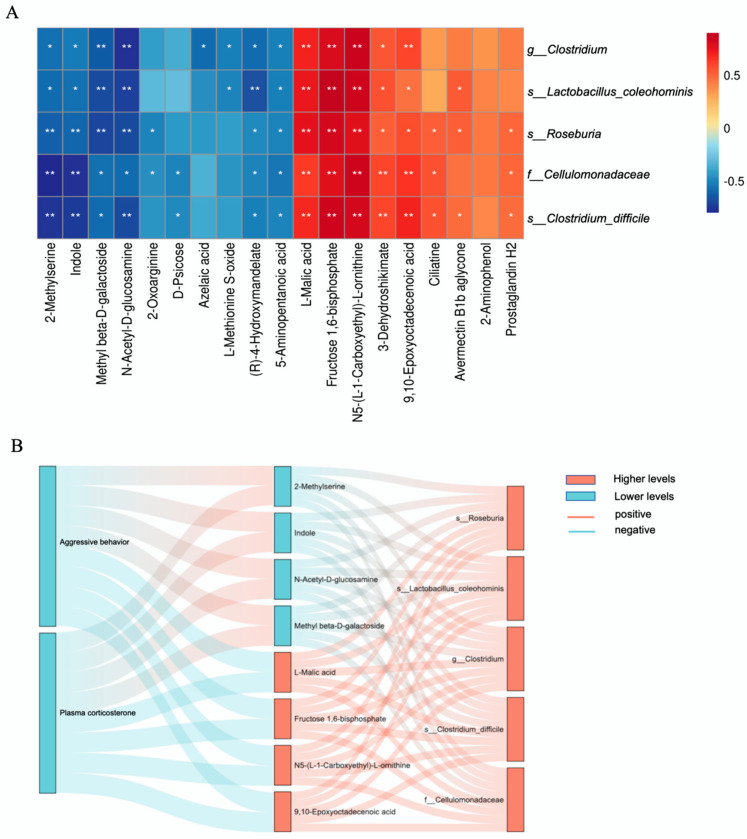
Specific differences correlation analysis in STI broilers. (**A**) Heatmap of top 5 specific microbiota in STI broilers and differential metabolites. (**B**) Sankey diagram of aggressive behavior, plasma corticosterone, top 5 microbiota, and differential metabolites in STI broilers. * *p* < 0.05, ** *p* < 0.01, n = 9.

To elucidate the potential interplay between cecal microbiota and metabolic regulation in aggressive behavior, we performed Spearman correlation analyses to assess the relationships between the top five dominant cecal microbial taxa and 19 differentially abundant metabolites in LTI broilers. As shown in Figure 6A, the six differential metabolites associated with aggressive behavior exhibited a strong correlation with the top five specific cecal microbiota in LTI broilers. Aggressive behavior and plasma corticosterone in LTI broilers were positively correlated with methyl beta-D-galactoside, N-acetyl-D-glucosamine, 5-aminopentanoic acid, and specific cecal microbiota, including *f_Prevotellaceae*, *s_Prevotellathe*, *f_Turicibacteraceae*, *s_Turicibacter*, and *s_Clostridium*; conversely, L-malic acid, N5-(L-1-carboxyethyl)-L-ornithine, and avermectin B1b aglycone showed a negative correlation with these behaviors and plasma corticosterone (Figure 6B). Eight main differential metabolites associated with aggressive behavior showed a strong correlation with the top five specific cecal microbiota in STI broilers, as shown in Figure 7A. Comprehensive analysis revealed distinct metabolite–microbiota–behavior associations in LTI and STI broilers. Aggressive behavior and plasma corticosterone levels exhibited significant positive correlations with 2-methylserine, indole, N-acetyl-D-glucosamine, and methyl β-D-galactoside, while these metabolites showed significant inverse associations with specific cecal microbiota, including *s_Roseburia*, *s_Lactobacillus_coleohominis*, *g_Clostridium*, *s_Clostridium_difficile*, and *f_Cellulomonadaceae*. Conversely, four additional metabolites—L-malic acid, fructose 1,6-bisphosphate, N5-(L-1-carboxyethyl)-L-ornithine, and 9,10-epoxyoctadecenoic acid—demonstrated negative correlations with aggressive behavior and corticosterone levels but positive associations with the top five dominant cecal microbiota in STI broilers (Figure 7B). Appearance biomarkers were positively correlated with higher-level metabolites in LTI broilers but negatively correlated in STI broilers. The higher-level metabolites in each group were positively correlated with specific cecal microbes in each group. These findings suggest a potential bidirectional relationship between microbial composition, metabolic profiles, and neuroendocrine regulation in mediating aggressive behavior in LTI broilers.

## 4. Discussion

This study revealed significant differences in intestinal morphology, cecal microbiota, and plasma metabolites among broiler chickens with distinct tonic immobility phenotypes. These findings highlight the complex interactions between physiological, microbial, and metabolic factors underlying aggressive behavior in poultry, offering novel insights into broiler welfare and management.

Aggressive behavior in broiler chickens is a critical welfare and productivity concern, as it negatively impacts feed conversion ratios, increases mortality rates, and compromises overall animal welfare. Our study demonstrated that broilers with short TI (STI) phenotypes exhibit lower plasma corticosterone (CORT) levels, reduced aggression, and better stress adaptability compared to LTI broilers. While this study found no performance differences between STI and LTI broilers under normal conditions, previous research indicates STI broilers exhibit better growth and stress adaptability under stressful conditions [17]. Elevated CORT levels in LTI broilers were positively correlated with increased aggression, corroborating prior research linking glucocorticoids to behavioral changes via stress-responsive pathways [18]. Corticosterone plays a crucial role in regulating various physiological processes, including metabolism and immune function [19,20], and its mobilization of energy reserves via gluconeogenesis and lipolysis links internal physiological states to stress responses [21]. Various factors, including feed restriction and environmental stress, can trigger aggression in broilers [22,23]. These findings emphasize the importance of CORT as a biomarker for behavioral and welfare traits in broilers. The lower plasma concentrations of AST, TP, Ca, and NEFA in STI broilers reflect their reduced stress responses and improved metabolic homeostasis compared to LTI broilers. Lower AST levels suggest better hepatic health and reduced oxidative stress, consistent with the attenuated corticosterone (CORT) levels observed in STI broilers [3,22]. Reduced plasma TP may indicate less protein catabolism due to diminished chronic stress, as elevated CORT in LTI broilers can stimulate gluconeogenesis and muscle protein breakdown [9,13]. Similarly, lower Ca levels in STI broilers may result from reduced stress-related calcium mobilization, which is often heightened in LTI broilers due to chronic HPA axis activation [8,14]. Decreased plasma NEFA levels in STI broilers likely reflect reduced lipolysis, a process driven by stress-induced glucocorticoid activity in LTI broilers. Together, these findings highlight the close relationship between stress physiology, metabolism, and biochemical profiles in broilers with distinct TI phenotypes.

The gut microbiome emerged as a critical regulator of aggressive behavior through its influence on the gut–brain axis [24]. Distinct TI phenotypes were associated with unique cecal microbiota compositions. Beneficial bacterial genera, such as Firmicutes, Bacteroidetes, and Lactobacillus, were enriched in STI broilers, contributing to the production of short-chain fatty acids (SCFAs) and neurotransmitter precursors like serotonin (5-HT) and dopamine (DA) [25,26]. SCFAs are known to modulate behavior by interacting with the enteric nervous system and immune pathways. In contrast, LTI broilers exhibited higher abundances of *Turicibacteraceae* and *Prevotellaceae*, which are associated with psychiatric disorders and glucose metabolism. *Prevotellaceae* is an important target for distinguishing bipolar depression, bipolar mania, and severe depression and was directly related to the stronger aggressive behavior observed in the LTI-phenotype broiler chickens [27]. *Turicibacteraceae* has a strong correlation with Parkinson’s disease, major depressive disease, and other psychiatric diseases [28,29]. *Clostridium* added to broiler diets as a supplement could significantly improve the intestinal health and growth performance of broilers [30,31]. *Roseburi* is an SCFA-rich flora in the gut [32] and can reverse nerve injury and restore cognitive impairment [33]. Previous research indicates that modifications in the gut Firmicutes composition could impact emotional response and TI duration [34]. Our findings identified a significant correlation between aggressive behavior and a key bacterial genus, *cc115*, belonging to the Firmicutes phylum, known for its SCFA metabolite production and potential behavioral impact. These microbial profiles may underlie the stronger aggressive tendencies observed in LTI broilers, suggesting potential applications for microbiota-based strategies to enhance broiler welfare.

Plasma metabolites reflected both microbial activity and host metabolic states, further linking gut microbiota to behavioral phenotypes. The decrease in indole production in broiler chickens leads to an increase in Firmicutes abundance and an elevated production of SCFAs [35], aligning with the findings regarding Firmicutes and indole levels in this study. STI broilers showed elevated levels of R-4-hydroxymandelate and fructose 1,6-bisphosphate, which are associated with antioxidant functions and glycolytic pathways, respectively. (R)-4-hydroxymandelate serves as a marker of sexual maturity in chickens and is associated with antioxidant functions within the organism [36]. In this study, broiler chickens with the STI phenotype showed decreased levels of (R)-4-hydroxymandelate, which is positively correlated with aggressive behavior. Conversely, metabolites like 5-aminopentanoic acid and azelaic acid, which are linked to lipid metabolism and glucose regulation [37,38,39,40], were elevated in LTI broilers, potentially exacerbating stress responses and aggression. Notably, prostaglandin H2, a precursor for inflammatory mediators, was significantly lower in LTI broilers but positively correlated with aggression and CORT levels [41]. In addition, we found that fructose 1,6-diphosphate was significantly negatively correlated with aggressive behavior, and the content of fructose 1,6-diphosphate in STI broilers was significantly increased. These findings underscore the intricate relationship between metabolic pathways and behavioral regulation in broilers.

## 5. Conclusions

This study provides a comprehensive understanding of the physiological, microbial, and metabolic mechanisms underlying aggressive behavior in broiler chickens with different TI phenotypes. By identifying key microbial genera (*g_cc115*, *s_Clostridium_difficile*, *s_Roseburia*, etc.) and plasma metabolites (L-malic acid, 2-methylserine, fructose 1,6-bisphosphate, etc.) associated with aggressive behavior, this research offers potential targets for intervention. Incorporating probiotics or prebiotics tailored to modulate gut microbiota may serve as a practical strategy to reduce aggression and improve broiler welfare. Additionally, monitoring plasma metabolites could provide non-invasive biomarkers for early detection of stress and behavioral issues in poultry farming, as well as their application in large-scale farming systems to validate their practical utility.

## Data Availability

Data are contained within the article and Supplementary Materials.

## Supplementary Materials

The following supporting information can be downloaded at https://www.mdpi.com/article/10.3390/ani15040601/s1: Table S1. Assessment of aggressive behavior in broilers. Table S2. Effects of different TI phenotypes on the serum biochemical parameters of broiler chickens. Figure S1. Differential metabolite classification pie chart. Figure S2. Correlations between all differential microbiota, all differential metabolites and aggressive behavior. * *p* < 0.05, ** *p* < 0.01, n = 9.
