# Supplementary material for: Integrated Microbiome and Metabolomics Analysis Reveals Altered Aggressive Behaviors in Broiler Chickens Showing Different Tonic Immobility
Source: Animals (Basel). 2025 Feb 19;15(4):601. doi: 10.3390/ani15040601 (PMC11851396; doi:10.3390/ani15040601)
Supplement: Supplementary file 1 [file animals-15-00601-s001.zip › animals-3451855-supplementary.pdf]

Table S1 Assessment of aggressive behavior in broilers

| Types of aggressive behavior | Description |
|---|---|
| Peck | The aggressor pecked at another individual |
| Grab | The aggressor clawed at the neck and back of another individual with their claws |
| Twist | The invader twisted the neck and back of another individual with their claws |

Table S2 Effects of different TI phenotypes on the serum biochemical parameters of broiler chickens

| Parameters | LTI | STI | *P-value* |
|---|---|---|---|
| ALT (U/L) | 15.40±1.69 | 14.50±1.57 | 0.702 |
| AST (U/L) | 230.90±8.71 | 209.00±2.34 | 0.026 |
| AST/ALT | 16.14±1.31 | 16.15±1.88 | 0.994 |
| TP (g/L) | 46.58±1.68 | 42.50±0.90 | 0.046 |
| ALB (g/L) | 14.22±0.46 | 14.23±0.49 | 0.988 |
| TBA (μmol/L) | 3.92±054 | 3.72±0.39 | 0.767 |
| GLU (mmol/L) | 12.82±0.23 | 12.79±0.31 | 0.937 |
| Ca (mmol/L) | 3.04±0.04 | 2.85±0.05 | 0.005 |
| CHOL (mmol/L) | 3.55±0.06 | 3.50±0.19 | 0.800 |
| TG (mmol/L) | 1.09±0.02 | 1.08±0.02 | 0.812 |
| HDL-C (mmol/L) | 2.65±0.08 | 2.67±0.16 | 0.930 |
| LDL-C (mmol/L) | 0.74±0.06 | 0.67±0.05 | 0.412 |
| LDH (U/L) | 441.30±46.25 | 506.80±33.97 | 0.269 |
| NEFA (mmol/L) | 0.74±0.03 | 0.60±0.03 | 0.008 |

ALT Alanine aminotransferase, AST Aspartate aminotransferase, TP Total Protein, ALB Albumin, TBA Total bile acids, GLU Glucose, CHOL Cholesterol,TG Triglycerides, HDL-C High-density lipoprotein cholesterol, LDL-C Low-density lipoprotein cholesterol, LDH Lactatedehydrogenase, GLOB Globulins, NEFA Nonestesterified fatty acid.

Note: Data are represented as mean ± SEM, n = 10. Differences between groups were analysed by ANOVA test (different letters means significant differences; $P < 0.05$).

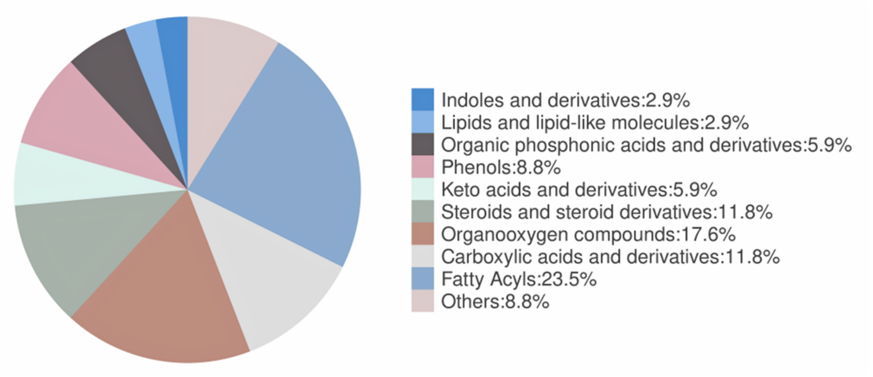


Figure S1 Differential metabolite classification pie chart.

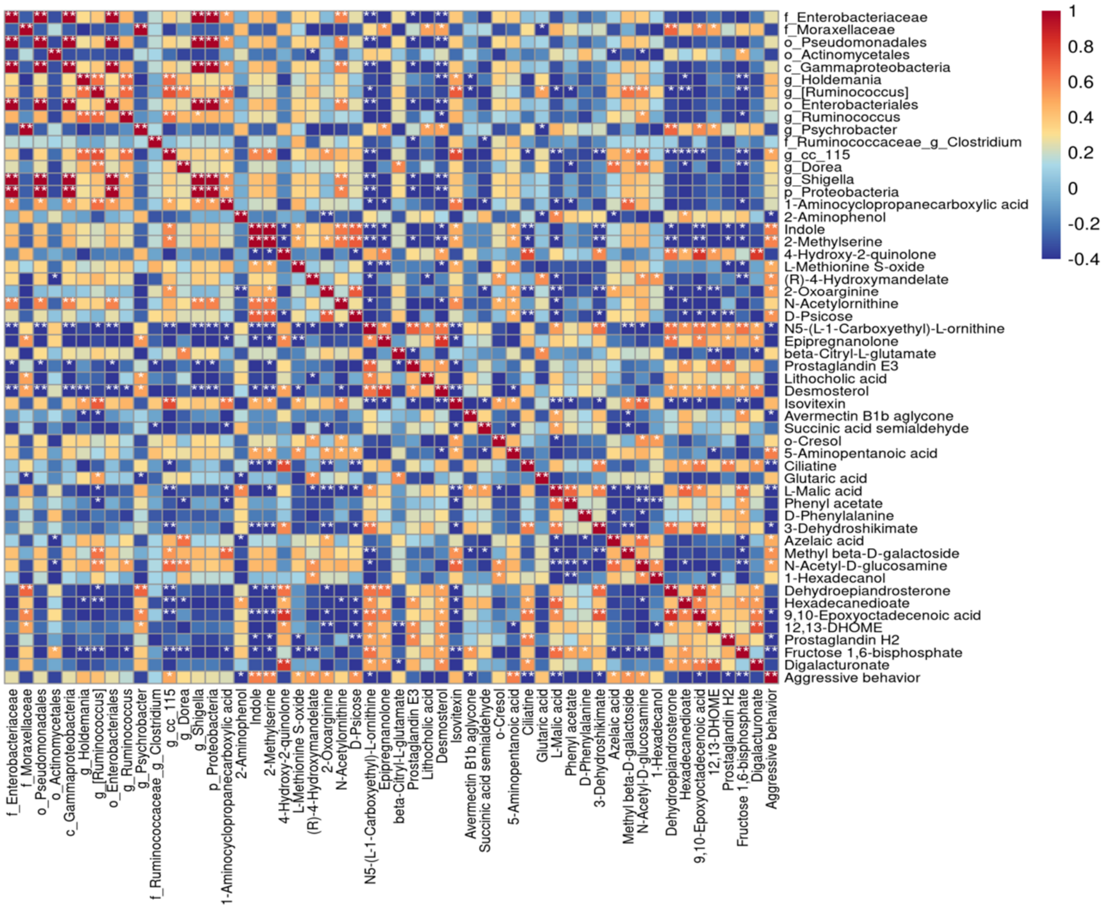


Figure S2 Correlations between all differential microbiota, all differential metabolites and aggressive behavior. ∗ $p < 0.05$, ∗∗ $p < 0.01$, n = 9.
